# Neurodevelopmental outcome of patients with congenital gastrointestinal malformations: a systematic review and meta-analysis

**DOI:** 10.1136/archdischild-2021-322158

**Published:** 2021-06-10

**Authors:** Daniëlle Roorda, Marsh Königs, Laurens Eeftinck Schattenkerk, Lideke van der Steeg, Ernest van Heurn, Jaap Oosterlaan

**Affiliations:** 1 Department of Pediatric Surgery, Amsterdam Reproduction and Development Research Institute, Emma Children's Hospital, Amsterdam UMC, University of Amsterdam and Free University Amsterdam, Amsterdam, The Netherlands; 2 Department of Pediatrics, Emma Neuroscience Group, Amsterdam Reproduction & Development Research Institute, Emma Children's Hospital, Amsterdam UMC, University of Amsterdam, Amsterdam, The Netherlands; 3 Pediatric Surgery, Princess Maxima Center for Pediatric Oncology, Utrecht, The Netherlands

**Keywords:** gastroenterology, neuropathology, psychology, neonatology

## Abstract

**Aim:**

Children with congenital gastrointestinal malformations may be at risk of neurodevelopmental impairment due to challenges to the developing brain, including perioperative haemodynamic changes, exposure to anaesthetics and postoperative inflammatory influences. This study aggregates existing evidence on neurodevelopmental outcome in these patients using meta-analysis.

**Method:**

PubMed, Embase and Web of Science were searched for peer-reviewed articles published until October 2019. Out of the 5316 unique articles that were identified, 47 studies met the inclusion criteria and were included. Standardised mean differences (Cohen’s d) between cognitive, motor and language outcome of patients with congenital gastrointestinal malformations and normative data (39 studies) or the studies’ control group (8 studies) were aggregated across studies using random-effects meta-analysis. The value of (clinical) moderators was studied using meta-regression and diagnostic subgroups were compared.

**Results:**

The 47 included studies encompassed 62 cohorts, representing 2312 patients. Children with congenital gastrointestinal malformations had small-sized cognitive impairment (d=−0.435, p<0.001; 95% CI −0.567 to −0.302), medium-sized motor impairment (d=−0.610, p<0.001; 95% CI −0.769 to −0.451) and medium-sized language impairment (d=−0.670, p<0.001; 95% CI −0.914 to −0.425). Patients with short bowel syndrome had worse motor outcome. Neurodevelopmental outcome was related to the number of surgeries and length of total hospital stay, while no relations were observed with gestational age, birth weight, age and sex.

**Interpretation:**

This study shows that children with congenital gastrointestinal malformations exhibit impairments in neurodevelopmental outcome, highlighting the need for routine screening of neurodevelopment during follow-up.

**What is already known on this topic?**

- Patients with non-cardiac congenital malformations are at risk of motor and cognitive impairment up to the age of 2 years.
- Patients with gastrointestinal malformations are subject to several potential aetiological factors contributing to negative impact on the developing brain.

**What this study adds?**

- Patients with congenital gastrointestinal malformations have impaired neurodevelopmental outcome up to adolescence.
- Cognitive impairment was small-sized, whereas motor and language impairment was medium-sized.
- Impairment was related to length of hospital stay and number of surgeries.

## Introduction

Congenital gastrointestinal malformations (ie, oesophageal atresia, gastroschisis, omphalocele, intestinal atresia, Hirschsprung’s disease and anorectal malformations) are relatively uncommon conditions with a total prevalence of about 15 per 10 000 European births a year.^1^ Although survival in these patients has improved over the past decades, morbidity remains high.^2–8^ Recent evidence suggests that there may also be an impact on the central nervous system of these patients.^9^

The available literature provides evidence for several pathways implicated in congenital gastrointestinal malformations that may contribute to a negative impact on the developing central nervous system: (1) genetic abnormalities^10^ ^11^; (2) perinatal influences, such as maternal smoking,^12^ use of medication,^13 14^ preterm birth^15^ and low birth weight^15^; (3) early, long and/or repeated exposure to anaesthetics necessary for surgical correction(s)^16–18^; (4) perioperative haemodynamics and respiratory functioning^19–21^; (5) postoperative inflammatory challenges^22 23^; and (6) poor nutritional status that can lead to an altered microbiome, influencing the developing brain through the gut–brain axis.^24–27^ All these harmful challenges to the central nervous system may lead to neurodevelopmental impairment, which in turn may interfere with development in important domains of functioning, including academic achievement, behavioural functioning, and social and economic well-being.^28–30^

The primary aim of the current systematic review is to quantitatively aggregate all available empirical evidence on the effects of having a congenital gastrointestinal malformation on neurodevelopment using meta-analysis. This review focuses on congenital gastrointestinal malformations other than congenital diaphragmatic hernia (CDH), to not include the confounding effect of the pulmonary comorbidity in patients with CDH,^31–33^ which may require treatment with extracorporeal membrane oxygenation.^34 35^ The secondary aim is to study differences between specific types of congenital gastrointestinal malformations and the contribution of possible moderating factors for neurodevelopmental impairment, using meta-regression.

## Methods

This study was performed according to the Preferred Reporting Items for Systematic Reviews and Meta-Analyses guidelines (see online supplemental material).^36^

10.1136/archdischild-2021-322158.supp1 Supplementary data

### Search and selection

The search strategy combined three groups of search terms and their equivalents: (1) terms related to the congenital malformations of interest, (2) terms defining age groups, (3) terms defining (the validated measures of) the outcomes. The full search strategy can be found in the online supplemental material. PubMed, Embase and Web of Science were searched using both simple search terms and hierarchical family forms (eg, Medical Subject Headings, Thesaurus, Emtree). The reference lists of eligible articles were also screened for additional articles. The last search was conducted in October 2019.

**Figure 1:**
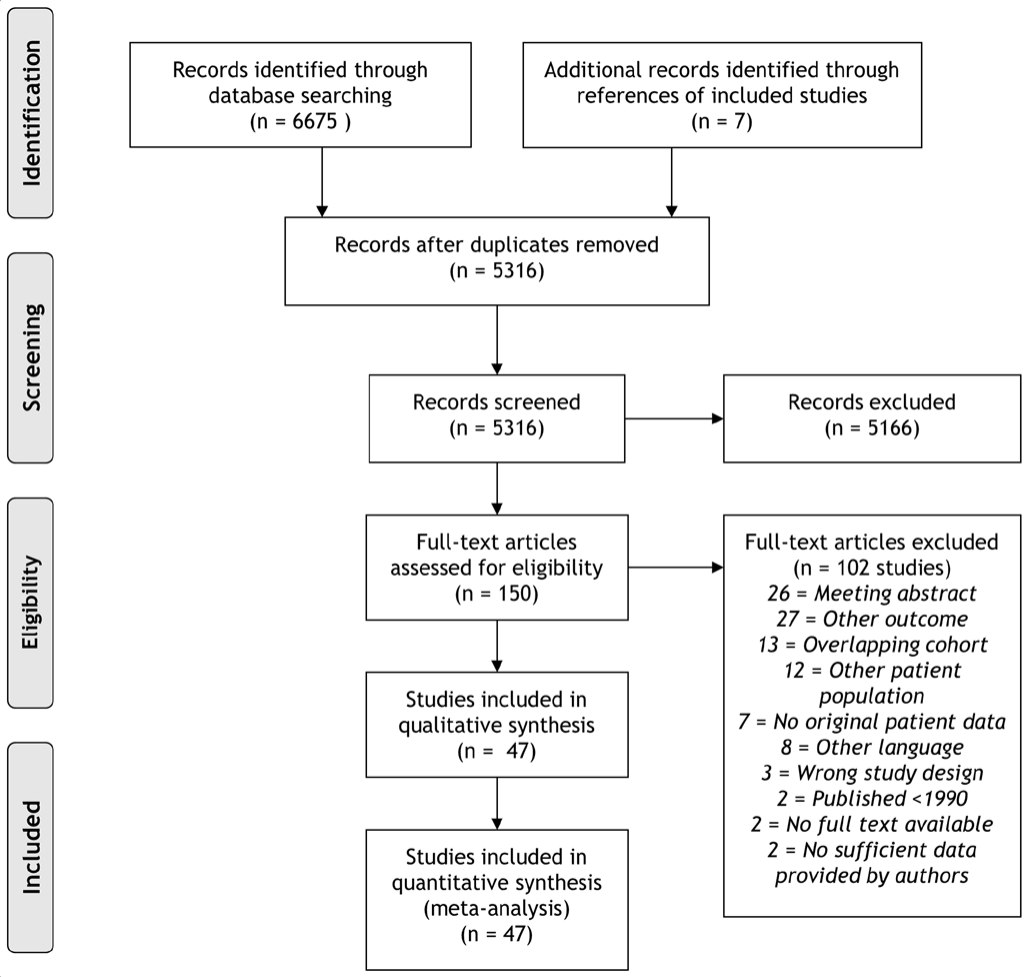
Preferred Reporting Items for Systematic Reviews and Meta-Analyses flow chart.

A flow diagram of the study search and selection is provided in figure 1. A total of 6675 records were identiﬁed corresponding to 5316 unique articles. Two authors (DR and LES) independently assessed each article for eligibility using Covidence, an online tool for systematic reviews.^37^ Conflicts in the selection process were solved by consensus, or a third party was consulted.

### Inclusion criteria

Studies were included in this systematic review and meta-analysis if they: (1) included patients with a congenital gastrointestinal malformation (ie, oesophageal atresia, omphalocele, gastroschisis, intestinal atresia, Hirschsprung’s disease, anorectal malformations and short bowel syndrome), excluding CDH, (2) included subjects within the age range from infancy to adolescence (0–18 years), (3) reported cognitive, motor or language outcome measured with any standardised and/or validated measure, compared with a selected control group or normative population, (4) used an observational or controlled design, (5) were published in a peer-reviewed journal, (6) were published after 1990, and (7) were written in the English language. Studies reporting on adults only, or studies reporting on both children and adults without detailing the results for only children, were excluded, as well as review papers and case reports. A cohort was defined as a subgroup of the total group of patients included in a study, mostly defined in terms of a particular congenital malformation, and in few studies defined in terms of age at follow-up. How selection was done in case multiple studies reported on (partly) overlapping cohorts is described in the online supplemental material. Authors of studies were contacted in case a study did not report all data required for the planned analyses. In total, 47 studies were included in the meta-analysis. A reference list is provided in the online supplemental material.

### Data extraction

The following data were extracted by two authors (DR and LES): (1) mean raw or standardised scores, accompanying SDs and sample sizes for all outcome measures were extracted for all separate cohorts of cases and, if applicable, control groups. If this information was not available, the proportion of individuals with neurodevelopmental outcome in the standardised normal range was compared between patients and the normative or control sample, in which case sample sizes and relevant p values were extracted. (2) Study characteristics, including: sample sizes, type(s) of malformation(s) assessed, instrument(s) used to assess neurodevelopment, length of follow-up, attrition of the study sample at follow-up; and (3) potential (clinical) moderating factors of neurodevelopmental outcome (listed in online supplemental material tables 1 and 3).

### Quality assessment

Quality of the included studies was independently assessed by two authors (DR and LES) using the Newcastle–Ottawa Scale (NOS), based on selection of subjects (4 points), comparability of patient and control groups (2 points) and outcome measurements (3 points).^38^ ^39^ Adjustments to the tool according to the manual and scoring methods are described in the online supplemental material. Rating discrepancies were resolved by consensus.

### Statistics

Analyses were performed using Comprehensive Meta-Analysis (CMA) software (V.3.0, Biostat). Using the extracted mean (SD) of raw or standardised scores on cognitive, motor and/or language outcome of cases, and of controls (8 studies) or normative data (39 studies), we calculated effect sizes as the standardised mean difference (Cohen’s d) between groups. Outcome measures in the current meta-analyses were: overall neurodevelopmental outcome and three domains of neurodevelopmental outcome: cognitive outcome, motor outcome and language outcome. Overall neurodevelopmental outcome was calculated on a study level by using the built-in function of CMA, which generates the weighted average of study findings across domains (motor, cognitive and/or language outcome). The individual study’s effect sizes were subsequently aggregated across studies into meta-analytical effect sizes using the random-effects model to account for heterogeneity introduced by the included range of outcome measures, diagnostic subgroups and age groups. These analyses were rerun, excluding studies that may have included subjects with chromosomal abnormalities, to eliminate the influence of neurodevelopmental impairment related to a syndrome. In case of statistical significant difference in overall neurodevelopmental outcome, differences between the meta-analytical findings for cognitive, motor and language outcome were further explored using subgroup comparisons. If a meta-analytical effect size was built up by a minimum of 10 individual studies’ effect sizes, we explored possible moderating effects on the outcomes using univariate meta-regression with a random-effects model. For cohorts that were assessed at multiple assessment points, a weighted average was calculated for the moderator variables at study level and used in meta-regression to calculate the relationship with the weighted average effect sizes of outcome data (Cohen’s d) at study level. When studies reported the median with IQRs, means and SD were calculated.^40 41^ Furthermore, subgroup comparisons were performed to test for possible differences between diagnostic subgroups (ie, different types of malformations) and domains of outcome. Effect sizes were interpreted as small (d=0.2–0.5) medium (d=0.5–0.8) or large (d≥0.8), according to Cohen.^42^ Heterogeneity was interpreted as small (I^2^≤0.25), medium (I^2^=0.25–0.50) or strong (I^2^≥0.50), according to Higgins.^43^ The possibility of publication bias was assessed by visual inspection of Funnel plots and by calculating Funnel plot asymmetry expressed as the Egger’s regression intercept t.^44^ To test for bias caused by studies with a fair or poor quality of design, sensitivity analyses were conducted on studies of good quality only.

## Results

### Sample description

This systematic review and meta-analysis represents a total of 2312 patients described in 47 studies (online supplemental table 1). A detailed sample description in terms of distribution of types of malformation, sex, age groups, birth weight and gestational age can be found in the online supplemental material.

### Overall neurodevelopmental outcome

**Figure 2:**
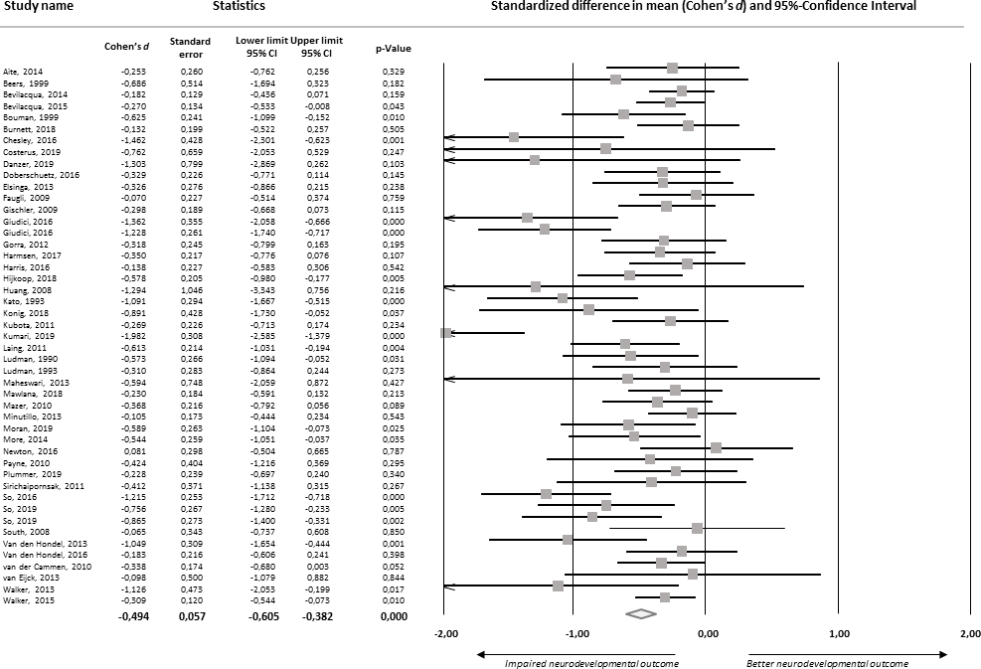
Forest plot of standardised mean differences in overall neurodevelopmental outcome.

The meta-analysis on overall neurodevelopmental outcome included all 47 studies (n=2312 patients; figure 2). Nineteen of the 47 studies showed significantly poorer overall neurodevelopmental outcome of patients with congenital gastrointestinal malformations compared with normative data or healthy controls. Meta-analytical aggregation of all findings showed a small-sized negative effect on overall neurodevelopmental outcome (d=−0.494, p<0.001; 95% CI −0.605 to −0.382, I^2^=56.2%; table 1).

**Table 1:** Meta-analytical findings for neurodevelopment in children with congenital gastrointestinal malformations

	Number of studies	Number of observations	Cohen’s d (95% CI, p value)	Difference between findings on domains of neurodevelopmental outcome	Heterogeneity, I^2^	Significant moderators	Egger’s intercept
Overall neurodevelopmental outcome	47	2312	−0.494 (−0.605 to −0.382, p<0.001)		56.2%	Mean length of stay: b*=*−0.005, p<0.001, mean number of surgeries: b=−0.1371, p=0.003	−1.874, p<0.001
*Cognitive outcome*	39	2055	−0.435 (−0.567 to −0.302, p=<0.001)	Q=3.194, p=0.343	63.7%	Mean number of surgeries: b=−0.0825, p=0.045	−0.711, p*=*0.031
*Motor outcome*	33	1821	−0.610 (−0.769 to −0.451, p=<0.001)		70.3%	Mean length of stay: b*=*−0.005, p=0.008, mean number of surgeries: b=−0.1789, p=0.001	−2.502, p*<*0.001
*Language outcome*	14	701	−0.670 (−0.914 to −0.425, p=<0.001)		68.4%	–	−2.743, p=0.013

### Cognitive outcome

The meta-analysis on cognitive outcome included 39 studies (n*=*2055 patients). In 12 of the 39 studies, cognitive outcome of patients with congenital gastrointestinal malformations was significantly worse compared with normative data or healthy controls. Meta-analytical aggregation showed a small-sized negative effect on cognitive outcome (d=−0.435, p<0.001; 95% CI −0.567 to −0.302, I^2^=63.7%; table 1).

### Motor outcome

The meta-analysis on motor outcome included 33 studies (n=1821 patients). In 14 of 33 studies, motor outcome of patients with congenital gastrointestinal malformation was significantly worse compared with the normative population or healthy controls. Meta-analytical aggregation showed a medium-sized negative effect on motor outcome (d=−0.610, p<0.001; 95% CI −0.769 to −0.451, I^2^=70.3%; table 1).

### Language outcome

The meta-analysis on language outcome included 14 studies (n*=*701 patients). Ten out of 14 studies showed a significant negative difference between language development of patients with a congenital gastrointestinal malformation and the normative population or healthy controls. Meta-analytical aggregation showed a medium-sized negative effect (d=−0.670, p<0.001; 95% CI −0.914 to −0.425, I^2^=68.4%; table 1).

### Influence of possible presence of chromosomal abnormalities

Sensitivity analyses excluding three studies that may have included subjects with chromosomal abnormalities showed comparable (if not larger) impairments on overall neurodevelopmental outcome (d*=*−0.519, p<0.001), cognitive outcome (d*=*−0.458, p<0.001), motor outcome (d*=*−0.658, p<0.001) and language outcome (d*=*−0.780, p<0.001).

### Meta-regression of possible moderators of neurodevelopmental outcome

Meta-regression showed that worse overall neurodevelopmental and worse motor outcome were related to longer mean total length of hospital stay, worse overall neurodevelopmental, worse cognitive and worse motor outcome were related to a higher mean number of surgeries, while no relations were observed with mean age, mean gestational age, mean birth weight and percentage of boys in a study, as shown in table 1 and online supplemental table 2.

### Differences between types of malformations

When comparing meta-analytical effect sizes of subgroups of different types of malformations, we found a significant difference in the magnitude of effect sizes for overall neurodevelopment outcome (Q=11.52; p=0.021) (table 2), that was traced down in further analyses to significantly poorer overall neurodevelopmental outcome for patients with short bowel syndrome compared with all remaining patient groups (d=−1.000 and d=−0.412, respectively, Q=11.639; p=0.001). Further tests assessing differences between types of malformations are shown in the online supplemental material.

**Table 2:** Differences between types of malformations in overall neurodevelopmental outcome

Type of malformation	Number of studies	Cohen’s d (95% CI, p value) on overall neurodevelopmental outcome	Type of malformation versus other types of malformation, Q-values, p values
Abdominal wall defects (ie, gastroschisis, omphalocele)	17	−0.375 (−0.567 to −0.182, p<0.001)	Q=1.14, p=0.286
Colorectal malformations (ie, Hirschsprung’s disease, anorectal malformations)	10	−0.485 (−0.765 to −0.206, p=0.001)	Q=0.024, p=0.877
Oesophageal atresia	17	−0.521 (−0.713 to −0.328, p<0.001)	Q=0.433, p=0.506
Intestinal atresia	5	−0.251 (−0.585 to −0.082, p=0.140)	Q=1.657, p=0.190
Short bowel syndrome	6	−1.000 (−1.324 to −0.675, p<0.001)	Q*=*11.639, p=0.002

### Quality of studies and risk of bias analysis

Results of the quality assessment are presented in table 3. NOS scores ranged from 4 to 9. Most studies had good quality (77%), with only a minority of studies qualifying as fair (15%) or poor (8%). Results of the sensitivity analysis on studies of good quality, risk of publication bias (see also table 1) and risk of other bias analyses are described in the online supplemental material.

**Table 3:** Quality of included studies as assessed with the Newcastle–Ottawa Scale (NOS)

Study	Selection of subjects	Comparability of cases and controls	Outcome measurements	Total score*	Quality†
Aite, 2014^57^	3	1	2	6	Good
Beers, 2000^25^	4	2	3	9	Good
Bevilacqua, 2014^46^	3	1	3	7	Good
Bevilacqua, 2015^58^	3	1	2	6	Good
Bouman, 1999^65^	3	1	2	6	Good
Burnett, 2018^66^	3	1	2	6	Good
Chesley, 2016^26^	3	1	3	7	Good
Costerus, 2019^67^	3	1	2	6	Good
Danzer, 2019^62^	3	1	2	6	Good
Doberschuetz, 2016^68^	4	2	3	9	Good
Elsinga, 2013^49^	3	1	2	6	Good
Faugli, 2009^69^	2	1	2	5	Fair
Gischler, 2009^8^	3	1	3	7	Good
Giudici, 2016^70^	3	0	3	6	Poor
Giudici, 2016^71^	3	0	2	5	Poor
Gorra, 2012^72^	3	2	2	7	Good
Harmsen, 2017^59^	3	1	3	7	Good
Harris, 2016^73^	3	1	2	6	Good
Hijkoop, 2017^74^	3	1	3	7	Good
Huang, 2008^22^	3	1	3	7	Good
Kato, 1993^60^	2	1	3	6	Fair
Konig, 2018^75^	2	1	3	6	Fair
Kubota, 2011^47^	2	1	2	5	Fair
Kumari, 2019^76^	3	0	1	4	Poor
Laing, 2011^77^	1	1	3	5	Poor
Ludman, 1990^61^	3	2	3	8	Good
Ludman, 1993^78^	3	2	3	8	Good
Maheshwari, 2013^79^	3	1	3	7	Good
Mazer, 2010^3^	3	1	3	7	Good
Mawlana, 2018^80^	3	1	3	7	Good
Minutillo, 2013^81^	3	1	3	7	Good
Moran, 2019^82^	3	2	2	7	Good
More, 2014^83^	3	1	2	6	Good
Newton, 2016^84^	4	2	2	8	Good
Payne, 2010^85^	4	2	3	9	Good
Plummer, 2019^86^	2	1	2	5	Fair
Sirichaipornsak, 2011^87^	3	1	2	6	Good
So, 2016^88^	3	1	3	7	Good
So, 2019^89^	2	1	2	5	Fair
So, 2019^90^	2	1	3	6	Fair
South, 2008^91^	3	1	3	7	Good
Van den Hondel, 2013^92^	3	1	3	7	Good
Van den Hondel, 2016^93^	3	1	3	7	Good
Van der Cammen-van Zijp, 2010^8^	3	1	2	6	Good
Van Eijck, 2013^50^	3	1	2	6	Good
Walker, 2013^94^	3	2	3	8	Good
Walker, 2015^95^	3	2	2	7	Good

*The NOS allows study quality of observational studies to be quantified on the basis of the methods used to select subjects (4 points), comparability of case and control groups (2 points) and outcome measurements (3 points).

†Scores were converted to the Agency for Healthcare Research and Quality standards, in order to judge quality as ‘good’, ‘fair’ or ‘poor’.

## Discussion

This systematic review and meta-analysis of 47 studies representing 2012 patients revealed evidence for small-sized overall neurodevelopmental impairment in children with congenital gastrointestinal malformations compared with normative data or healthy controls, reflecting small-sized cognitive impairment, medium-sized motor impairment and medium-sized language impairment. These findings translate into an average difference in 6.5 IQ points and implicate a 3.6% increase in the number of children with cognitive delay, a 5.9% increase in the number of children with motor delay and 6.9% increase in the number of children with language delay. Excluding studies that may have included syndromal patients did not lead to altered conclusions. Our findings implicate that patients with congenital gastrointestinal malformations have increased risk of neurodevelopmental impairment. Our findings are in line with an earlier meta-analysis of cognitive and motor impairment in infants (up to 24 months of age) with non-cardiac congenital malformations,^45^ although the slightly larger effects obtained in that meta-analysis may be explained by the inclusion of patients with CDH.^46^

Robust evidence for neurodevelopmental impairment was found in all types of congenital gastrointestinal malformations. Contrary to what has been indicated in previous reports,^46 47^ no differences in meta-analytical effect sizes of overall neurodevelopmental outcome were found between patients with specific types of congenital gastrointestinal malformations, except for relatively poorer overall neurodevelopment and motor development in patients with short bowel syndrome.

Considering moderating factors, the results revealed that longer mean length of stay and a higher mean number of surgeries were related to greater overall neurodevelopmental impairment and motor development. This may suggest that the more complex the course of disease and/or treatment that is required, the more profound the impact is on neurodevelopmental outcome. The results of our meta-regression analyses showed no differences in the magnitude of effect between the different age groups. This cross-sectional finding suggests that the magnitude of neurodevelopmental impairment remains relatively stable over developmental stages, but remains to be investigated by longitudinal studies.

Although preterm birth and low birth weight are associated with neurodevelopmental impairment,^26 48–54^ meta-regression analyses found no evidence for the possibility that our findings reflect the effects of gestational age or birth weight. This suggests that other common aetiological factors for neurodevelopmental impairment may play a (more important) role in the neurodevelopmental impairments of patients with congenital gastrointestinal malformations, such as factors related to intrauterine development,^55 56^ surgical treatment,^19 26 57–62^ compromised bowel function and feeding support,^50 57 62^ and parental social economic status.^63^ We consider this an important issue in future research and suggest prospective registration of potential aetiological factors and neurodevelopment outcomes.

The evidence found in this meta-analysis was primarily based on studies with good quality (74%). Excluding studies with fair or poor quality did not result in altered conclusions. Risk of publication bias analyses indicated a potential influence of publication bias on the meta-analytical estimations, indicating that these estimations should be interpreted with caution and emphasising importance for preregistration of study protocols.

### Limitations

The findings of the current systematic review and meta-analysis are limited by the use of normative data in the majority of included studies (38 of 47), which does not control for differences in variables such as sex and socioeconomic status. Second, there was heterogeneity in the measures used to assess neurodevelopmental outcome, while some evidence suggests that the Bayley Scales of Infant Development (BSID)-III may overestimate neurodevelopment as compared with the BSID-II.^64^ Third, the tests of subgroup differences on type of malformations and type of outcome domain were limited by partially overlapping subjects across subgroups. However, since related observations tend to decrease variance, this would make the comparison more sensitive for group differences, which were not observed. Fourth, the quantity of available literature allowed inclusion of only a limited number of potentially moderating aetiological factors in meta-regression and was subject to distinct heterogeneity in terms of construct definitions. Lastly, due to the limited number of studies, our findings for language outcome and the possible influence of moderating factors on all outcomes await replication before a firm conclusion may be drawn.

## Conclusions and clinical implications

In conclusion, this systematic review and meta-analysis presents robust evidence that patients with congenital gastrointestinal malformations are at risk of small-sized to medium-sized impairment in neurodevelopmental outcome, emphasising the need for routine neurodevelopmental screening of these patients.

## Data Availability

Data are available upon reasonable request.
