# Supplementary material for: Neurodevelopmental outcome of patients with congenital gastrointestinal malformations: a systematic review and meta-analysis
Source: Arch Dis Child Fetal Neonatal Ed. 2021 Jun 10;106(6):635–42. doi: 10.1136/archdischild-2021-322158 (PMC8543204; doi:10.1136/archdischild-2021-322158)
Supplement: Supplementary data [file archdischild-2021-322158supp001.pdf]

Supplementary Material

SEARCH STRATEGY PUBMED

("Congenital Abnormalities"[Mesh:NoExp] OR congenital abnormalit*[tiab] OR congenital deformit*[tiab] OR congenital defect*[tiab] OR birth defect*[tiab] OR congenital anomal*[tiab] OR "Esophageal Atresia"[Mesh] OR esophageal atresia*[tiab] OR oesophageal atresia*[tiab] OR "Gastroschisis"[Mesh] OR gastroschis*[tiab] OR Congenital Fissure of the Abdominal Cavity[tiab] OR ("Hernia, Umbilical"[Mesh] AND congenital*[tiab]) OR exomphalos[tiab] OR omphalocele*[tiab] OR "Hirschsprung Disease"[Mesh] OR hirschsprung disease[tiab] OR congenital megacolon[tiab] OR hirschsprung's disease[tiab] OR hirschsprungs disease[tiab] OR aganglionic megacolon[tiab] OR Rectosigmoid Colon Aganglionosis[tiab] OR Rectosigmoid Aganglionosis[tiab] OR Congenital Intestinal Aganglionosis[tiab] OR Colonic Aganglionosis[tiab] OR Total Colonic Aganglionosis[tiab] OR "Anorectal Malformations"[Mesh] OR Anorectal Malformation*[tiab] OR Anorectal Anomal*[tiab] OR Anorectal Atresia*[tiab] OR Anorectal Stenos*[tiab] OR "Anus, Imperforate"[Mesh] OR imperforate anus[tiab] OR anal atresi*[tiab] OR "Short Bowel Syndrome"[Mesh] OR Short Bowel Syndrome*[tiab] OR intestinal failure[tiab] OR pediatric intestinal failure[tiab] OR paediatric intestinal failure[tiab] OR "Intestinal Atresia"[Mesh] OR Congenital Intestinal Atresia*[tiab] OR Apple Peel Syndrome*[tiab] OR Apple-Peel Intestinal Atresia*[tiab] OR Jejunal Atresia[tiab] OR Apple Peel Small Bowel Syndrome[tiab] OR Familial Apple Peel Jejunal Atresia[tiab]) AND (Infan*[tiab] OR newborn*[tiab] OR new-born*[tiab OR perinat*[tiab] OR neonat*[tiab] OR baby[tiab] OR baby*[tiab] OR babies[tiab] OR toddler*[tiab] OR minors[tiab] OR minors*[tiab] OR boy[tiab] OR boys[tiab] OR boyfriend[tiab] OR boyhood[tiab] OR girl*[tiab] OR kid[tiab] OR kids[tiab] OR child[tiab] OR child*[tiab] OR children*[tiab] OR schoolchild*[tiab] OR schoolchild[tiab] OR school child[tiab] OR school child*[tiab] OR adolescen*[tiab] OR juvenil*[tiab] OR youth*[tiab] OR teen*[tiab] OR under*age*[tiab] OR pubescen*[tiab] OR pediatrics[mesh] OR pediatric*[tiab] OR paediatric*[tiab] OR peadiatric*[tiab] OR school[tiab] OR school*[tiab] OR prematur* OR preterm*) AND ("Child Development"[Mesh] OR Child Development[tiab] OR Infant Development[tiab] OR neurocogniti*[tiab] OR neuropsych*[tiab] OR cogniti*[tiab] OR neurodevelopment*[tiab] OR developmental[tiab] OR motor*[tiab] OR movement[tiab] OR psychomotor[tiab] OR intell*[tiab] OR intellect*[tiab] OR intellectual[tiab] OR intelligence[tiab] OR psychomotor performanc*[tiab] OR neurocognitive performanc*[tiab] OR psychomotor skil*[tiab] OR neurocognitive skil*[tiab] OR neuropsychological funct*[tiab] OR psychomotor funct*[tiab] OR neuropsychological outcom*[tiab] OR psychomotor outcom*[tiab] OR neurocognitive outcom*[tiab] OR "Wechsler Scales"[Mesh] OR Wechsler Scale*[tiab] OR WPPSI[tiab] OR WISC[tiab] OR Wechsler Intelligence Scale for Children[tiab] OR Wechsler Preschool and Primary Scale of

Intelligence[tiab] OR BSID[tiab] OR Bayley[tiab] OR Bayley Scales of Infant Development[tiab] OR MABC[tiab] OR Movement Assessment Battery for Children[tiab] OR AIMS[tiab] OR Alberta Infant Motor Scale[tiab] OR Infant Motor Scale[tiab] OR BOTMP[tiab] OR Bruininks-Oseretsky[tiab] OR Bruininks*[tiab] OR Griffiths[tiab] OR Griffiths Mental Development Scale[tiab] OR GMSD[tiab] OR Griffith score*[tiab] OR Mullen[tiab] OR Mullen Scales of Early Learning[tiab] OR MSEL[tiab] OR Ages and stages questionnaire[tiab] OR ASQ[tiab] OR CBCL[tiab] OR Child Behavioural Checklist[tiab] OR Child Behavioral Checklist[tiab] OR NEPSY[tiab]) AND ("Case-Control Studies"[Mesh] OR "Cohort Studies"[Mesh] OR "Observational Study" [Publication Type] OR case-control[tiab] OR cohort[tiab] OR retrospective[tiab] OR prospective[tiab] OR observational stud*[tiab] OR descriptive study[tiab] OR "Cross-Sectional Studies"[Mesh] OR cross sectional[tiab])

SELECTION OF STUDIES DESCRIBING OVERLAPPING COHORTS

In case multiple articles reported on (partly) overlapping cohorts, we included the article that: (1) reported the longest follow-up period, (2) reported separately on subgroups of patients to allow differentiation for malformation type and/or (3) had the largest sample size to maximize generalizability of the sample and statistical power of meta-analysis (decisions were made in this order).

ADJUSTED USE OF THE NEWCASTLE OTTAWA SCALE FOR QUALITY ASSESSMENT OF INCLUDED STUDIES

In accordance with the manual, the NOS tool was adjusted to enable quality assessment of observational cohort studies. One aspect of the scale ("demonstration that outcome of interest was not present at start of the study") was not applicable and was therefore omitted from quality assessment. An extra point for selection was given when studies had a design with a control group instead of using normative scores.

In accordance with the Agency for Healthcare Research and Quality (AHRQ) standards, quality of studies was considered:

- 'good', in case of a score of 3-4 points for selection of subjects AND a score of 1-2 points for comparability of cases and controls AND a score of 2-3 points for outcome measurements
- 'fair', in case of a score of 2 points for selection of subjects AND a score of 1-2 points for comparability of cases and controls AND a score of 2-3 points for outcome measurements
- 'poor', in case of a score of 0-1 points for selection of subjects OR 0 for comparability of cases and controls OR 0-1 for outcome measurements

REFERENC LIST OF INCLUDED STUDIES

Aite L, Bevilacqua F, Zaccara A, et al. Short-term neurodevelopmental outcome of babies operated on for low-risk esophageal atresia: a pilot study. Diseases of the Esophagus. 2014;27(4):330-334.

Beers SR, Yaworski JA, Stilley C, Ewing L, Barksdale EM. Cognitive deficits in school-aged children with severe short bowel syndrome (SBS). Pediatrics. 1999;104(3):770-770.

Bevilacqua F, Morini F, Valfre L, et al. Surgical gastrointestinal anomalies including diaphragmatic hernia: Does type of anomaly affect neurodevelopmental outcome? American journal of perinatology. 2014;31(3):175-180.

Bevilacqua F, Rava L, Valfre L, et al. Factors affecting short-term neurodevelopmental outcome in children operated on for major congenital anomalies. Journal of Pediatric Surgery. 2015;50(7):1125-1129.

Bouman NH, Koot HM, Hazebroek FW. Long-term physical, psychological, and social functioning of children with esophageal atresia. Journal of pediatric surgery. 1999;34(3):399-404.

Burnett AC, Gunn JK, Hutchinson EA, et al. Cognition and behaviour in children with congenital abdominal wall defects. Early human development. 2018;116:47-52.

Chesley PM, Sanchez SE, Melzer L, et al. Neurodevelopmental and Cognitive Outcomes in Children With Intestinal Failure. Journal of Pediatric Gastroenterology and Nutrition. 2016;63(1):41-45.

Costerus S, Vlot J, van Rosmalen J, Wijnen R, Weber F. Effects of Neonatal Thoracoscopic Surgery on Tissue Oxygenation: A Pilot Study on (Neuro-) Monitoring and Outcomes. European journal of pediatric surgery. 2019;29:166-72.

Danzer E, Hoffman C, Miller JS, D'Agostino JA, Schindewolf EM, Gerdes M, et al. Autism spectrum disorder and neurodevelopmental delays in children with giant omphalocele. Journal of pediatric surgery. 2019;54:1771-7.

Doberschuetz N, Dewitz R, Rolle U, Schlosser R, Allendorf A. Follow-Up of Children with Gastrointestinal Malformations and Postnatal Surgery and Anesthesia: Evaluation at Two Years of Age. Neonatology. 2016;110(1):8-13.

Elsinga RM, Roze E, Van Braeckel KN, Hulscher JB, Bos AF. Motor and cognitive outcome at school age of children with surgically treated intestinal obstructions in the neonatal period. Early human development. 2013;89(3):181-185.

Faugli A, Bjornland K, Emblem R, Novik TS, Diseth TH. Mental health and psychosocial functioning in adolescents with esophageal atresia. Journal of pediatric surgery. 2009;44(4):729-737.

Gischler SJ, Mazer P, Duivenvoorden HJ, et al. Interdisciplinary structural follow-up of surgical newborns: a prospective evaluation. Journal of pediatric surgery. 2009;44(7):1382-1389.

Giudici L, Bokser VS, Maricic MA, Golombek SG, Ferrario CC. Babies born with gastroschisis and followed up to the age of six years faced long-term morbidity and impairments. Acta Paediatrica. 2016;105(6):E275-E280.

Giudici LB, Bokser VS, Golombek SG, Castrillon CC, Trovato M, Ferrario CC. Esophageal atresia: long-term interdisciplinary follow-up. Journal of Pediatric and Neonatal Individualized Medicine. 2016;5(2).

Gorra AS, Needelman H, Azarow KS, Roberts HJ, Jackson BJ, Cusick RA. Long-term neurodevelopmental outcomes in children born with gastroschisis: the tiebreaker. Journal of pediatric surgery. 2012;47(1):125-129.

Harmsen WJ, Aarsen FJ, van der Cammen-van Zijp MHM, et al. Developmental problems in patients with oesophageal atresia: A longitudinal follow-up study. Archives of Disease in Childhood. 2017;102: F214–F219.

Harris EL, Hart SJ, Minutillo C, et al. The long-term neurodevelopmental and psychological outcomes of gastroschisis: A cohort study. Journal of Pediatric Surgery. 2016;51(4):549-553.

Hijkoop A, H IJ, Wijnen RMH, Tibboel D, Rosmalen JV, Cohen-Overbeek TE. Prenatal markers and longitudinal follow-up in simple and complex gastroschisis. Archives of disease in childhood Fetal and neonatal edition. 2018;103(2):F126-F131.

Huang J, Cai W, Tang Q, et al. Long-term cognitive functions in neonatal short bowel syndrome patients. European Journal of Pediatric Surgery. 2008;18(2):89-92.

Kato T, Kanto K, Yoshino H, et al. Mental and intellectual development of neonatal surgical children in a long-term follow-up. Journal of pediatric surgery. 1993;28(2):123-129.

Konig TT, Muensterer OJ. Physical Fitness and Locomotor Skills in Children With Esophageal Atresia-A Case Control Pilot Study. Front Pediatr. 2018;6:337.

Kubota A, Nose K, Yamamoto E, et al. Psychosocial and cognitive consequences of major neonatal surgery. Journal of pediatric surgery. 2011;46(12):2250-2253.

Kumari V, Joshi P, Dhua AK, Sapra S, Srinivas M, Agarwala S, et al. Developmental Status of Children Operated for Esophageal Atresia with or without Tracheoesophageal Fistula Along with Maternal Stress, Their Quality of life, and Coping Abilities at AIIMS, New Delhi. European journal of pediatric surgery. 2019;29:125-31.

Laing SR, Walker K, Ungerer J, Badawi N, Spence K. Early development of children with major birth defects requiring newborn surgery. Journal of Paediatrics and Child Health. 2011;47(3):140-147.

Ludman L, Spitz L, Lansdown R. Developmental progress of newborns undergoing neonatal surgery. Journal of pediatric surgery. 1990;25(5):469-471.

Ludman L, Spitz L, Lansdown R. Intellectual development at 3 years of age of children who underwent major neonatal surgery. Journal of pediatric surgery. 1993;28(2):130-134.

Maheshwari R, Trivedi A, Walker K, Holland AJ. Retrospective cohort study of long-gap oesophageal atresia. Journal of paediatrics and child health. 2013;49(10):845-849.

Mawlana W, Zamiara P, Lane H, et al. Neurodevelopmental outcomes of infants with esophageal atresia and tracheoesophageal fistula. Journal of pediatric surgery. 2018;53(9):1651-1654.

Mazer P, Gischler SJ, MH VDC-VZ, et al. Early developmental assessment of children with major non-cardiac congenital anomalies predicts development at the age of 5 years. Developmental medicine and child neurology. 2010;52(12):1154-1159.

Minutillo C, Rao SC, Pirie S, McMichael J, Dickinson JE. Growth and developmental outcomes of infants with gastroschisis at one year of age: A retrospective study. Journal of Pediatric Surgery. 2013;48(8):1688-1696.

Moran MM, Gunn-Charlton JK, Walsh JM, Cheong JLY, Anderson PJ, Doyle LW, et al. Associations of Neonatal Noncardiac Surgery with Brain Structure and Neurodevelopment: A Prospective Case-Control Study. The Journal of pediatrics. 2019;212:93-101 e2

More K, Rao S, McMichael J, Minutillo C. Growth and Developmental Outcomes of Infants with Hirschsprung Disease Presenting in the Neonatal Period: A Retrospective Study. Journal of Pediatrics. 2014;165(1):73-+.

Newton LE, Abdessalam SF, Raynor SC, et al. Neurodevelopmental outcomes of tracheoesophageal fistulas. Journal of Pediatric Surgery. 2016;51(5):743-747.

Payne NR, Gilmore L, Svobodny S, et al. A cross-sectional, case-control follow-up of infants with gastroschisis. Journal of Neonatal-Perinatal Medicine. 2010;3(3):207-215.

Plummer EA, Wang Q, Larson-Nath CM, Scheurer JM, Ramel SE. Body composition and cognition in preschool-age children with congenital gastrointestinal anomalies. Early human development. 2019;129:5-10.

Sirichaipornsak S, Jirapradittha J, Kiatchoosakun P, Suphakunpinyo C. Neurodevelopmental outcomes of children with gastroschisis at university hospital in northeast Thailand. Asian Biomedicine. 2011;5(6):861-866.

So S, Patterson C, Gold A, et al. Early neurodevelopmental outcomes of infants with intestinal failure. Early Human Development. 2016;101:11-16.

So S, Patterson C, Evans C, Wales PW. Motor Proficiency and Generalized Self-Efficacy Toward Physical Activity in Children With Intestinal Failure. Journal of pediatric gastroenterology and nutrition. 2019;68:7-12.

So S, Patterson C, Gold A, Rogers A, Belza C, de Silva N, et al. Neurodevelopmental outcomes of infants with intestinal failure at 12 and 26months corrected age. Early human development. 2019;130:38-43.

South AP, Marshall DD, Bose CL, Laughon MM. Growth and neurodevelopment at 16 to 24 months of age for infants born with gastroschisis. Journal of Perinatology. 2008;28(10):702-706.

van den Hondel D, Aarsen FK, Wijnen RM, Sloots CE, H IJ. Children with congenital colorectal malformations often require special education or remedial teaching, despite normal intelligence. Acta paediatrica (Oslo, Norway : 1992). 2016;105(2):e77-84.

van den Hondel D, Sloots CE, Gischler SJ, Meeussen CJ, Wijnen RM, H IJ. Prospective long-term follow up of children with anorectal malformation: growth and development until 5years of age. Journal of pediatric surgery. 2013;48(4):818-825.

van der Cammen-van Zijp MH, Gischler SJ, Mazer P, van Dijk M, Tibboel D, Ijsselstijn H. Motor-function and exercise capacity in children with major anatomical congenital anomalies: an evaluation at 5 years of age. Early human development. 2010;86(8):523-528.

van Eijck FC, van Vlimmeren LA, Wijnen RM, et al. Functional, motor developmental, and long-term outcome after the component separation technique in children with giant omphalocele: a case control study. Journal of pediatric surgery. 2013;48(3):525-532.

Walker K, Halliday R, Badawi N, Stewart J, Holland AJ. Early developmental outcome following surgery for oesophageal atresia. Journal of paediatrics and child health. 2013;49(6):467-470.

Walker K, Loughran-Fowlds A, Halliday R, et al. Developmental outcomes at 3 years of age following major non-cardiac and cardiac surgery in term infants: A population-based study. Journal of Paediatrics and Child Health. 2015;51(12):1221-1225.

STUDY CHARACTERISTICS OF INCLUDED STUDIES

sTable 1 *Study characteristics of included studies*

**Author**	**Period of inclusion[a]**	**Country**	**Sample size[b]**	**Assessed type of malformation[b]**	**Tool[c]**	**Normative sample**	**Included in meta-analysis of overall neurodevelopmental outcome**	**Included in meta-analysis of motor outcome**	**Included in meta-analysis of cognitive outcome**	**Included in meta-analysis of language outcome**	**Age at assessment (months)[a,de,f]**	**Mean (SD) GA (wk)[a,d,f]**	**Mean (SD) BW (gram)[a,d,f]**	**Mean (SD) number of surgeries[a,d,e,f]**	**Mean (SD) length of total hospital stay[a,d,e,f]**
Aite, 2014	2008-2012	Italy	30	EA	BSID-III	Italian norm[1]	X	X	X		6 / 12	38 (2)	2635 (470)	1.1 (0.2)/ 1.2 (0.5)	NR
Beers, 2000	NR	USA	8	SB	WISC-III	North-American norm[2]	X	X	X		117	34 (3)	19889 (826)	NR	NR
Bevilacqua, 2014	2008-2010	Italy	150 (37 EA, 43	Mix	BSID-III	Italian norm[1]	X	X	X		6	38 (1)	2944 (636)	1.7 (1.5)	34.9 (23.2)

			CDH, 29 MM, 16 CAWD, 25 CR)												
			156 (38 EA, 40 CDH, 35 MM, 17 CAWD, 26 CR)	Mix	BSID-III	Italian norm[1]	X	X	X		12	38 (1)	2935 (643)	2 (1.5)	40.4 (30.7)
			84 (15 EA, 30 CDH, 19 MM, 12 CAWD, 8 CR)	Mix	BSID-III	Italian norm[1]	X	X	X		24	38 (2)	2853 (547)	2 (1.5)	40.9 (27.2)
Bevilacqua, 2015	2008-2012	Italy	41	EA	BSID-III	Italian norm[1]	X	X	X		6 / 12	38 (2)	2714 (553)	1.4 (0.8) / 1.4 (0.8)	36. 8 (27.7) / 39.4

															(30.7)
			34	IA	BSID-III	Italian norm[1]	X	X	X		6 / 12	37 (2)	2763 (667)	2 (1.5) / 2 (1.5)	56.0 (49.0) / 55.2 (48.8)
			18	GS, OM	BSID-III	Italian norm[1]	X	X	X		6 / 12	37 (2)	2614 (607)	1.4 (0.8) / 1.4 (0.8)	30.4 (9.5) / 30.4 (9.5)
			20	HD, ARM	BSID-III	Italian norm[1]	X	X	X		6 / 12	39 (2)	3326 (618)	2.3 (1.6) / 2.4 (1.8)	33.3 (26.1) / 34.5 (28.9)
Bouman, 1999	NR	Netherlands	36	EA	WISC-RN	Dutch norm[3]	X		X		122	NR	NR	3.4 (1.9)	NR
Burnett, 2018	2006-2014	Australia	39	GS	BSID-III	North-American norm[4]	X	X	X	X	2	36 (2)	2194 (400)	NR	39.6 (25.4)

			20	GS	WPPSI-III	North-American norm[5]			X		72	35 (2)	2304 (614)	NR	47.9 (41.9)
			20	OM	BSID-III	North American norm[4]	X	X	X	X	24	39 (2)	3351 (596)	NR	15.4 (14.3)
			10	OM	WPPSI-III	North American norm[5]			X		72	39 (1)	3492 (532)	NR	11.5 (7.7)
Chesley, 2016	NR	USA	15	SB	BSID-II	North American norm[6]	X	X	X		17	34 (4)	NR	11 (5.7)	145.9 (93.7)
Costerus, 2019	2011-2013	Netherlands	5	EA	BSID-II	Dutch norm[7]	X	X	X		24	38 (2)	2742 (545)	NR	NR
Danzer, 2019	2004-2015	USA	47	OM	BSID-II; BSID-III, WPPSI-III,	NR	X	X	X	X	41	35 (3)	2525 (735)	NR	132.8 (86.3)

					WPPSI-IV										
Doberschuetz, 2016	2008-2011	Germany	40 (9 EA, 9 GS, 5 IA, 4 OM, 3 CDH, 2 ARM, 1 HD, 3 combination)	Mix	BSID-II	Control group – but normative data was included	X	X	X		25	37 (2)	2782 (674)	2.0 (NR)	43.9 (40.3)
Elsinga, 2013	1995-2002	Netherlands	27	IA	M-ABC, WISC-III, NEPSY-II	Dutch norm[8,9,10]	X	X	X		114	36 (3)	2972 (1091)	NR	NR
Faugli, 2009	1999-2002	Norway	39	EA	BSID-II	North American	X	X	X		13	NR	2780 (926)	1.4 (0.9)	NR

						norm[11]									
Gischler, 2009	1999-2003	Netherlands	17	EA	BOS 2-30	Dutch norm[12]	X	X	X		6 / 12 / 18 / 24	39 (3)	2928 (485)	4.4 (4.0)	77.2 (78.8) / 79.8 (80.8) / NR / NR
			34	IA	BOS 2-30	Dutch norm[12]	X	X	X		6 / 12 / 18 / 24	37 (3)	2964 (697)	2 (1.5)	46.1 (44.3) / 49.0 (47.2) / NR / NR
			19	GS, OM	BOS 2-30	Dutch norm[12]	X	X	X		6 / 12 / 18 / 24	39 (3)	2744 (641)	2.3 (1.6)	48.2 (36.8) / 55.4 (52.9) / NR / NR
Giudici, 2016	2003-2013	Argentinia	27/14/13	EA	CATCLAMS /	North American	X	X	X	X	12 / 36 / 72	38 (2)	2917 (440)	NR	NR

					PRUNAPE	norm (Catclams),[13] Argentinian norm (Prunape)[14]									
Giudici, 2016	2003-2014	Argentinia	52/34/20	GS	CATCLAMS / PRUNAPE	North American norm (Catclams)[13] Argentinian norm (Prunape)[14]	X	X	X	X	12 / 36 / 72	37 (2)	2403 (427)	NR	NR
Gorra, 2012	2001-2008	USA	46	GS	BSID-II	Control group – but normative data was included	X				24	36 (NR)	2542 (NR)	NR	54.0 (NR
Harmsen, 2017	1999-2006	Netherlands	54	EA	MABC, MABC-II,	Dutch norm[8,15,9]	X	X	X		60 / 96	38 (3)	2798 (816)	NR / NR	84.6 (99.6) / NR

					WISC-III										
Harris, 2016	1992-2005	Australia	39	GS	WPPSI-III, WISC-IV	Australian norm[5,16]	X		X		120	36 (2)	2496 (174)	NR	NR
Hijkoop, 2017	2000-2012	Netherlands	54	GS	BOS 2-30, BSID-II	Dutch norm[12,7]	X	X	X		12 / 24	36 (2)	2313 (NR)	NR	NR
Huang, 2008	2005-2006	China	8	SB	BSID-I, WPPSI-R, WISC-R, WAIS-R	North American norm[17-20]	X	X	X		80	38 (1)	3291 (377)	NR	NR
Kato, 1993	1978-1983	Japan	8	GS, OM	WISC-R	NR	X		X		107	NR	NR	NR	NR
			6	HD	WISC-R	NR	X		X		101	NR	NR	NR	NR

			13	ARM	WISC-R	NR	X		X		94	NR	NR	NR	NR
Konig, 2018	NR	Germany	12	EA	KTT; DMT	Control group	X	X			84	NR	NR	NR	NR
Kubota, 2011	NR	Japan	20	EA	WISC-III	North American norm[21]	X		X		NR	NR	NR	NR	NR
			25	ARM	WISC-III	North American norm[21]	X		X		NR	NR	NR	NR	NR
Kumari, 2019	2012-2017	India	32	EA	DASII	Indian norm[22]	X		X		17	37 (3)	2360 (639)	NR	NR
Laing, 2011	2002-2004	Australia	46	Mix	BSID-II	North American norm[6]	X	X	X		24	38 (2)	3174 (578)	1.5 (0.9)	28.0 (20.8)
Ludman, 1990	1983-1984	UK	30	Mix	GMDS	Control group	X	X	X	X	12	NR	NR	NR	52.5 (65.6)
Ludman, 1993	1983-1985	UK	29	Mix	GMDS	Control group	X	X	X	X	6 / 36	NR	NR	NR / NR	NR / NR

Maheshwari, 2013	2006-2011	Australia	3	EA	BSID-III	NR	X	X	X	X	5-13	NR	NR	NR	NR
Mawlana, 2018	2000-2015	Canada	182	EA (TEF)	BSID-II	North American norm[4]	X	X	X	X	24	37 (3)	2589 (800)	NR	NR
Mazer, 2010	1999-2002	Netherlands	15	EA	BOS 2-30, MABC	Dutch norm[12,8]	X	X	X		6 / 12/ 24 / 60	NR	NR	NR / NR / NR / NR	NR / NR / NR / NR
			18	IA,	BOS 2-30, MABC	Dutch norm[12,8]	X	X	X		6 / 12/ 24 / 60	NR	NR	NR / NR / NR / NR	NR / NR / NR / NR
			27	GS, OM	BOS 2-30, MABC	Dutch norm[12,8]	X	X	X		6 / 12/ 24 / 60	NR	NR	NR / NR / NR / NR	NR / NR / NR / NR
			6	HD	BOS 2-30, MABC	Dutch norm[12,8]	X	X	X		6 / 12/ 24 / 60	NR	NR	NR / NR / NR / NR	NR / NR / NR / NR
			15	ARM	MABC, BOS 2-	Dutch norm[12,8]	X	X	X		60	NR	NR	NR	NR

					30										
Minutillo, 2013	1997-2010	Australia	67	GS	GMDS	NR	X		X		12	NR	NR	NR	NR
Moran, 2019	2011-2013	Australia	27 (10 EA, 17 CAWD)	Mix	BSID-III	Control group	X	X	X	X	26	38 (32-40)	2940 (NR)	NR	NR
More, 2014	2001-2010	Australia	31	HD	GMDS	British norm[23]	X		X		12	NR	NR	NR	NR
Newton, 2016	2001-2014	USA	34	EA (TEF)	BSID-II, BSID-III	Control group	X				35	35 (NR)	2244 (NR)	NR	57.7 (NR)
Payne, 2010	1999-2007	USA	57	GS	BSID-III	Control group	X				39	36 (NR)	2365 (NR)	NR	54.2 (42.6)
Plummer, 2019	2016-2018	USA	34	CGIA	NIH Toolbox	British norm[24]	X		X	X	56	38 (2)	3220 (690)	NR	NR
Sirichaipornsak, 2011	2007-2008	Thailand	15	GS	BSID-III	NR	X	X	X	X	22	37 (2)	2289 (477)	NR	42.4 (29.3)

So, 2016	2011-2013	Canada	33	SB	AIMS, MAI	North American norm[25,26]	X	X			11	33 (5)	1877 (1031)	2.4 (0.8)	165.5 (99.6)
So, 2019	2011-2013	Canada	30	SB	MSEL	North American norm[27]	X	X	X	X	12-15 / 26-32	33 (5)	1949 (995)	NR / NR	NR / NR
So, 2019	2015-2016	Canada	30	SB	BOT2	North American norm[28]	X	X			84	35 (5)	2198 (848)	3.6 (2.3)	198.0 (128.4)
South, 2008	2003-2005	USA	17	GS	BSID-II	North American norm[6]	X				20	36 (2)	2360 (731)	NR	NR
Van den Hondel, 2013	1999-2011	Netherlands	37	ARM	RAKIT, MABC	Dutch norm[29,8]	X	X			60	38 (NR)	3010 (NR)	3.9 (3.5)	NR
Van den Hondel, 2016	1999-2006	Netherlands	43	HD, ARM	WISC-III, RAKIT	Dutch norm[9,29]	X	X	X		96	NR	NR	NR	NR

Van der Cammen -van Zijp, 2010	1999-2003	Netherlands	29	EA	MABC	Dutch norm[8]	X	X			71	37 (3)	2839 (913)	NR	76.9 (69.3)
			25	IA	MABC	Dutch norm[8]	X	X			71	37 (3)	2747 (509)	NR	49.4 (51.2)
			24	GS, OM	MABC	Dutch norm[8]	X	X			71	38 (2)	2702 (591)		59.8 (64.5)
Van Eijck, 2013	2004-2007	Netherlands	8	OM	MABC-II	Dutch norm[15]	X	X			72	NR	NR	NR	NR
Walker, 2013	2006-2008	Australia	31	EA (TEF)	BSID-III	Control group	X	X	X	X	12	38 (NR)	2718 (717)	NR	31.1 (30.7)
Walker, 2015	2006-2008	Australia	124	Mix	BSID-III	Control group	X	X	X	X	36	NR	NR	NR	NR

[a]NR = not reported

[b]ARM = anorectal malformations; BA = biliary atresia; CGIA = Congenital Gastrointestinal Anomalies; EA = esophageal atresia; GS = gastroschisis; HD = Hirschsprungs disease; IA = intestinal atresia / midgut malformations ; NCCA = non-cardiac congenital malformations; OM = omphalocele; SB = short bowel syndrome / intestinal failure

[c]AIMS = Alberta Infant Motor Scales; BSID = Bayley Scales of Infant Development; CATCLAMS = cognitive adaptive test/clinical linguistic and auditory milestone scale; DASII = Developmental Assessment Scale of Indian Infants; DMT = Deutscher Motorik Test; GMDS = Griffiths Mental Development Scale; KTT = Kinderturntest; MABC = Movement Assessment Battery for Children; MELS = Mullen Early Learning Scales; PRUNAPE = prueba nacional de pesquisa, a national screening program in Argentinia; RAKIT = Revisie Amsterdamse Kinder Intelligentie Test (Dutch intelligence test); WAIS = Wechsler Adult Scale of Intelligence; WPPSI = Wechsler Prechool and Primary Scale of Intelligence; WISC = Wechsler Infant Scale of Intelligence

[d] Reported median (range) and median (IQR) were recalculated into Mean (SD) using http://www.math.hkbu.edu.hk/~tongt/papers/median2mean.html

[e] Repeated measures are indicated by a "/" in this table.

[f] For all moderator variables a weighted average was calculated and included in meta-regression.

References to normative data

1. Bayley N. Bayley Scales of Infant and Toddler Development, 3rd Edition, Administration Manual. Florence: Giunti OS, 2010.
2. Wechsler D:Wechsler Intelligence Scale for Children (ed 3). San Antonio, TX, Psychological Corporation, 1991
3. Van Haassen PP, De Bruyn EEJ, Pijl YL, et al: WISC-R, Wechsler Intelligence Scale for Children-Revised, Dutch Translation. Lisse, The Netherlands, Swets & Zeitlinger, 1986
4. N. Bayley, Bayley Scales of Infant and Toddler Development, Third Edition (Bayley-III), San Antonio, TX, Harcourt Assessment, 2006.
5. D. Wechsler, The Wechsler Preschool and Primary Scale of Intelligence, Third Edition Australian Standardised Edition (WPPSI-III Australian), Pearson, Sydney, NSW, 2004.
6. Bayley N. Bayley Scales of Infant Development, 2nd edn. San Antonio, TX: The Psychological Corporation, 1993.
7. Ruiter SAJ, Spelberg HCL, van der Meulen BF, Nakken H. The BSIDII NL: construction, standardisation, and instrumental utility. Neth J Psychol 2008;64(01):15–40
8. Smits-Engelsman BCM. Movement Assessment Battery for Children. Lisse, The Netherlands: Swets & Zeitlinger; 1998.
9. Kort W, Compaan EL, Bleichrodt N, Resing WCM, Schittekatte M, Bosmans M, et al. WISC-III NL Handleiding. Amsterdam: NIP dienstencentrum; 2002.
10. Korkman M, Kirk U, Kemp SL. NEPSY II Clinical and Interpretative Manual. A Brand of Harcourt Assessments. San Antonio, TX: PsychCorp. 2007.
11. Bayley, N. (1993). Bayley scales of infant development (2nd ed.). New York: Psychological Corporation.
12. Van der Meulen BF, Smrkovsky M. De Bayley Ontwikkelings Schalen (BOS2-30). Handleiding. Lisse, the Netherlands: Swets and Zeitlinger; 1983.
13. Visintainer P, Bennett A. Standardization of capute scales. In The Capute Scale. Cognitive Adaptive Test/Clinical Linguistic Auditory Milestone Scale (CAT/CLAMS), 2nd ed. Baltimore: Paul Brookes Publishing Co, 2005: 47–70.

14. Lejarraga H, Menéndez AM, Menzano E, Guerra L, Biancato S, Pianelli P, et al. PRUNAPE: screening for psychomotor development problems at primary care level. Arch Argent Pediatr 2008; 106: 119–25.
15. Smits Engelsman BC. Movement abc-2-nl. Dutch manual. Amsterdam: Pearson, 2010.
16. Wechsler D. Wechsler Intelligence Scale for Children - Fourth Edition Australian Standardised Edition (WISC-IV Australian). Pearson; 2005.
17. Bayley N. Manual for the Bayley Scales of Infant Development. San Antonio: The Psychological Corporation, 1969
18. Wechsler D. Manual for the Wechsler Preschool and Primary Scale of Intelligence-Revised. New York, NY: Psychological Corp., 1990
19. Wechsler D. Manual for the Wechsler Intelligence Scale for Children-Revised. New York, NY: Psychological Corp., 1974
20. Wechsler D. Manual for the Wechsler Adult Intelligence Scale-Revised (WAIS-R). New York, NY: Psychological Corp., 1981
21. Wechsler D. Wechsler Intelligence Scale for Children—Third Edition. Philadelphia, PA: The Psychological Corporation; 1991.
22. Phatak P. Manual for Using Developmental Assessment Scales for Indian Infants (DASII); Based on Revised Baroda Norms. Pune, India: Anand Agencies; 1997
23. Huntley M. The Griffiths Mental Development Scales from birth to 2 years Manual (Revision). Association for Research in Infant and Child Development; 1996.
24. J.L. Beaumont, R. Havlik, K.F. Cook, R.D. Hays, K. Wallner-Allen, S.P. Korper, et al., Norming plans for the NIH toolbox, Neurology 80 (Issue 11, Supplement 3) (2013) S87–S92.
25. M.C. Piper, J. D, Motor Assessment of the Developing Infant, WB Saunders, Philadephia, 1994.
26. L.S. Chandler, M.S. A, M.W. Swanson, Movement Assessment of Infants: A Manual (Seattle, Washington) 1980.
27. E.M. Mullen, Mullen Scales of Early Learning Item Administration Book, A ed., NCS, Pearson, 1995.
28. Bruininks RH, Bruininks BD. BOT2 Bruininks-Oseretsky Test of Motor Proficiency. 2nd ed. Circle Pines, MN: AGS Publishing; 2005.
29. Bleichrodt N, Drenth PJD, Zaal JN, et al. Revisie Amsterdamse Kinder Intelligentie Test. Instructie, normen, psychometrische gegevens. Amsterdam: Pearson; 1984.

SAMPLE DESCRIPTION

Seventeen studies reported on a cohort of patients with one single malformation, whereas most studies reported on multiple subgroups of different malformations (30 studies), together resulting in a total of 62 cohorts: esophageal atresia (17 cohorts, n=603 patients), congenital abdominal wall defects (18 cohorts, n=585 patients), followed by colorectal malformations (10 cohorts, n=172 patients), intestinal atresia (5 cohorts, n=131 patients) and intestinal failure (4 cohorts, n=102 patients) and a combination of different congenital malformations (8 cohorts, n=486 patients). Neurodevelopmental outcomes in infants or toddlers (up to 36 months of age) were described in 29 studies (n=1,768 patients), whereas in 21 studies (n=794 patients) neurodevelopmental outcomes of children (up to a maximum age of 13 years) were described. Mean birthweight of patients ranged from 1,980 to 3,492 grams, with 13 studies reporting on cohorts with a mean birthweight < 2500 grams. Mean gestational age of patients ranged from 34 to 39 weeks, with 22 studies reporting on cohorts with a mean gestational age below 37 weeks. The mean proportion of males represented was 55% (ranging from 29% to 80%).

RESULTS OF META-REGRESSION ANALYSES

The following potential moderating factors were assessed in the current meta-analysis: mean age at neurodevelopmental testing, mean gestational age, mean birthweight, sex, comorbidity, growth impairment, neurological complications, age at primary surgery, number of surgeries, number of anesthetic exposures, length of total hospital stay, mean days of mechanical ventilation, mean days of parental nutrition, educational level of parents and socio-economic status of parents.

sTable 2 *Moderating effects of studies' effect sizes of studies' mean age, mean gestational age, mean birthweight and proportion of males on overall neurodevelopmental outcomes, cognitive outcomes and motor outcomes*

	**Overall neurodevelopmental outcomes**		**Cognitive outcomes**		**Motor outcomes**		Language outcomes[b]
	Number	*Significance*	*Number*	*Statistical*	*Number*	*Statistical*	

	of patients	*effect in meta-regression*	*of patients*	*effect in meta-regression*	*of patients*	*effect in meta-regression*	
Mean age at testing in months	n=1182	p=0.43	n=847	p=0.29	n=880	p=0.94	*NA*
Mean gestational age in weeks	n=1260	p=0.07	n=1155	p=0.12	n=1014	p=0.10	*NA*
Mean birthweight in grams	n=1255	p=0.08	n=1194	p=0.07	n=1009	p=0.45	*NA*
Sex[a]	n=790	p=0.24	n=608	p=0.20	n=545	p=0.45	*NA*
Mean total length of hospital stay in days	n=1098	p<0.001	n=931	p=0.24	n=817	p=0.008	*NA*
Mean number of surgeries	n=983	P=0.003	n=920	p=0.04	n=834	p=0.001	*NA*
a. Sex was expressed as the percentage of male subjects in each cohort<br>b. NA= not asessed							

sTable 3 *Potential moderating factors not assessed in meta-regression*

**Author**	**Comorbidity**	**Growth**	**Times exposure to anesthesia**	**Age at 1st surgery in days**	**Neurologic complications**	**Feeding**	**Ventilation**	**Parental education**	**Parental SES**
Aite		*n (%) weight <5th percentile*					*Median (range) number of days ventilation*	*n (%) by categories (below high school, high school, degree) by type of parent*	*n (%) by categories (salariate, intermediate, working class, unemployed) by type of parent*
Beers		*n by categories of percentile weight scores (<5th, 5-10th, 10-25th, 25-50th, 75th)*							
Bevilacqua	*n (%) associated malformations (1, more than 1)*							*n (%) by categories (primary school, secondary school, high school, degree) by type of parent*	
Bevilacqua	*n (%) associated malformations (none, 1, more than 1)*		*Median (IQR) by follow-up duration*		*n intracranial hemorraghe*	*n (%) medical appliances for feeding*	*median (IQR) ventilatory time in hours n (%) medical appliances for respiratory*	*n by categories (primary school, secondary school, high school, degree) by type of parent*	*n by categories (class 1-4) by type of parent*
Bouman							*Proportion assisted ventilation*		*Categorial (mid, low, high) by type of parent*
Burnett	*n (%) chromosomal abnormality*	*n (%) small for gestational age at birth (<10th)*		*Median (IQR) in days by ype malformation and age*		*n (%) discharged with tube feeding*		*n (%) low maternal education*	*n (%) receiving government assistence*

**Author**	**Comorbidity**	**Growth**	**Times exposure to anesthesia**	**Age at 1st surgery in days**	**Neurologic complications**	**Feeding**	**Ventilation**	**Parental education**	**Parental SES**
Chesley					*n with cerebral palsy*	*n with TPN; Median (range) number of days exposed to PN*			
Costerus	*n with comorbidity*			*Median (range) in days*			*Median (range) number of days ventilation*		
Danzer				*Median (range) in days*		*Mean (SD) age at initial feeding*	*Median (range) number of days ventilation*	*n (%) maternal education by categories (none, parttime, fulltime)*	*n (%) maternal education by categories (high school, partly college/college degree, graduate degree)*
Doberschuetz	*n (%) combined malformations*		*Mean (95%CI) duration in hours*	*Mean (95%CI) in hours*	*n with hearing impairment*	*Mean (SD) / median (range) number of days PN, Mean (SD) / Median (range) number of days tube feeding*	*Mean (SD) / median ((range) number of hours ventilation, Mean (SD) / Median (range) number of days oxygen*		*mean score (low, medium, high)*
Elsinga	*n (%) late onset sepsis or BPD*	*median weight*		*Median (range) in days*					
Faugli	*n with associated malformations*					*n (%) with feeding difficulties*	*n (%) assisted ventilation*	*median (range) number of years of maternal education*	

Author	Comorbidity	Growth	Times exposure to anesthesia	Age at 1st surgery in days	Neurologic complications	Feeding	Ventilation	Parental education	Parental SES
Gischler	*n (%) additional medical problems, n (%) septic complications, n (%), median (IQR) number of congenital anomalis*				*n (%) neurologic complications*	*n (%) NG tube at home, n (%) enterostomy at home*	*n (%) tracheostomy, n (%) oxygen at home*		*n (%) by categories (low, middle, high)*
Giudici		*n (%) weight <10th percentile*			*n with cerebral palsy; n with hearing loss*	*Mean (SD) / Median (IQR) number of days PN*	*Mean (Sd) and median (IQR) number of days assisted ventilation*		
Giudici		*n (%) weight <10th percentile*			*n with cerebral palsy; n with hearing loss*	*Mean (SD) / Median (IQR) number of days PN*	*Mean (Sd) and median (IQR) number of days assisted ventilation*		
Gorra									
Harmsen	*n (%) sepsis; n (%) vacterl-association; n (%) cardiac anomaly*		*Median (range) in hours, median (range) number of anesthetic exposures*			*Median (range) number of days PN*	*Median (range) number of days ventilation*		*n (%) by categories (low, middle, high)*
Harris					*n with amblyopia; n with cerebral palsy; n with hearing loss*				

**Author**	**Comorbidity**	**Growth**	**Times exposure to anesthesia**	**Age at 1st surgery in days**	**Neurologic complications**	**Feeding**	**Ventilation**	**Parental education**	**Parental SES**
Hijkoop	*n (%) multiple congenital anomalies, n (%) complications*	*n (%) SGA at birth*	*Median (IQR) procedures under GA*			*n (%) with intestinal failure; Median (IQR) number of days to full enteral feeding*	*Median (IQR) number of days ventilation by type of malformation*		*median (IQR) maternal SES score, n (%) low maternal SES score*
Huang		*weight for age Z-score per subject*		*age in days per subject*		*Duration of PN in days per subject*			
Kato									
Konig		*n (%) weight for age Z-score < 2*							
Kubota									
Kumari	*n (%) associated congenital anomalies*	*n (%) -3SD weight for age*					*n (%) on mechanical ventilation*	*n (%) graduated mothers*	*n (%) homemakers and monthly family income*
Laing				*Median (IQR) in days*	*n (%) with microcephaly*		*Median (IQR) hours assisted ventilation*	*n (%) by categories (<12y schooling, >12y, tertiary or further, bachelor or higher degree) by type of parent*	n (%) occupation father (skilled, unskilled, associate professional, professional)
Ludman							*n (%) assisted ventilation >4 days*		*n (%) by categories (manual, non-manual, single mom)*
Ludman									
Maheswari									

**Author**	**Comorbidity**	**Growth**	**Times exposure to anesthesia**	**Age at 1st surgery in days**	**Neurologic complications**	**Feeding**	**Ventilation**	**Parental education**	**Parental SES**
Mawlana	*n (%) VACTERL, n (%) associated anomalies, n (%) chromosomal anomalies*	*n (%) weight <10th percentile*				*n (%) with gastrostomy*			
Mazer	*Median (IQR) number of congenital anomalies, n (%) syndromal / chromosomal abnormality*					*Median (IQR) medical appliances (O2 or tracheostomy) at discharge*	*Median (IQR) medical appliances (NG tube or enterostomy) at discharge*		
Minutillo									
Moran					*MRI abnormalities*			*n (%) maternal tertiairy education*	*n (%) higher social risk*
More				*Median (IQR) age in days*					
Newton							*Mean days on ventilator*		
Payne		*% weight <10th percentile*				*n (%) all per oris feeding at discharge*	*Mean (SD) ventilator days*		*n (%) single mother*
Plummer		*weight for age z score*				*Median (IQR) number of days PN; Percentage PN <>7days; Percentage gastrostomy*	*Median (IQR) days of assisted ventilation; Percentage >2 days assisted ventilation*	*n (%) maternal eductation (college or higher)*	
Sirichaipornsak									

**Author**	**Comorbidity**	**Growth**	**Times exposure to anesthesia**	**Age at 1st surgery in days**	**Neurologic complications**	**Feeding**	**Ventilation**	**Parental education**	**Parental SES**
So					*n with cerebral palsy*	*Median (IQR) number of days PN first year; Percentage patients 100% full enteral feeding first year*			
So		*weight for age Z-score*			*n with microcephaly*	*Median (IQR) number of days PN; % PN dependance at follow-up; % enterostomy*			
So					*n with CNS comorbidity; n with cerebral palsy; n with hearing loss; n with visual comorbidity*	*Median (IQR) number of days PN first year/ first two years; n (%) PN at follow-up of two years; n (%) 100% full enteral feeds at follow-up of two years*			
South		*% weight <10th percentile*			*n (%) abnormal neurological exam*	*Mean (SD) number of days to full enteral feedings; mean (sd) number of days PN*	*Mean (SD) number of days assisted ventilation*		

Author	Comorbidity	Growth	Times exposure to anesthesia	Age at 1st surgery in days	Neurologic complications	Feeding	Ventilation	Parental education	Parental SES
Van den Hondel 2013	*n (%) at least 1 major associated anomaly; n with suspected (not diagnosed) syndrome*	*n (%) Small for gestational age at birth*				*n (%) gastrostomy*			
Van den Hondel	*n (%) major comorbidity present*		*Median (IQR) by type of malformation; Median (IQR) by follow-up duration*						*n (%) by categories (low, middle, high) by type of malformation*
Van der Cammen-van Zijp	*n (%) associated malformations*	*n (%) Small for gestational age at birth*					*Median (range) days ventilation support, n (%) ECMO*		
Van Eijck	*n with congenital tethered spinal cord syndrome*								
Walker	*n (%) of associated malformations*								
Walker									
Reason for exclusion from univariate meta regression	***Heterogeneity of definitions***	***Heterogeneity of definitions***	***Not enough studies***	***Not enough studies***	***Heterogeneity of definitions***	***Too biased by type of malformation***	***Too biased by type of malformation***	***Heterogeneity of definitions***	***Heterogeneity of definitions***

RESULTS OF SUBGROUP ANALYSES ON TYPE OF MALFORMATION

Motor outcomes were significantly different across subgroups of malformations (Q=11.704, p=0.020). Cognitive (Q=3.798, p=0.434) and language outcomes (Q=0.589, p=0.745) did not significantly differ across different types of malformations.

Further analyses showed that patients with short bowel syndrome had significantly worse motor outcomes compared to all remaining patient groups (d=-1.062 and d=-0.474, Q=7.682; p=0.006), but comparable cognitive outcomes (d=-0.241 and d=-0.432,Q=2.875; p=0.09) and language outcomes (d=-0.692 and d=-0.598, Q=0.038; p=0.85).

RESULTS OF SENSITIVITY ANALYSIS OF STUDIES OF GOOD QUALITY

Effect sizes for overall neurocognitive outcomes and cognitive outcomes differed between studies of different quality ratings (Q=21.46, p<0.001 and Q=45.53, p<0.001, respectively). Sensitivity analysis on studies of good quality (n=36), showed that the reported meta-analytic findings (impairments) were replicated for overall neurodevelopmental outcomes (d=-0.371,95%CI: -0.462 – 0.280, p<0.001), cognitive outcomes (d=-0.281, 95%CI: -0.363 - -0.199, p<0.001), motor outcomes (d=-.0568, 95%CI: -0.738 - -0.398, p<0.001) and language outcomes (d=-0.570, 95%CI: -0.865 - -0.274, p<0.001).

RISK OF BIAS ANALYSIS

sFigure1 *Funnelplot of overall neurodevelopmental outcomes*

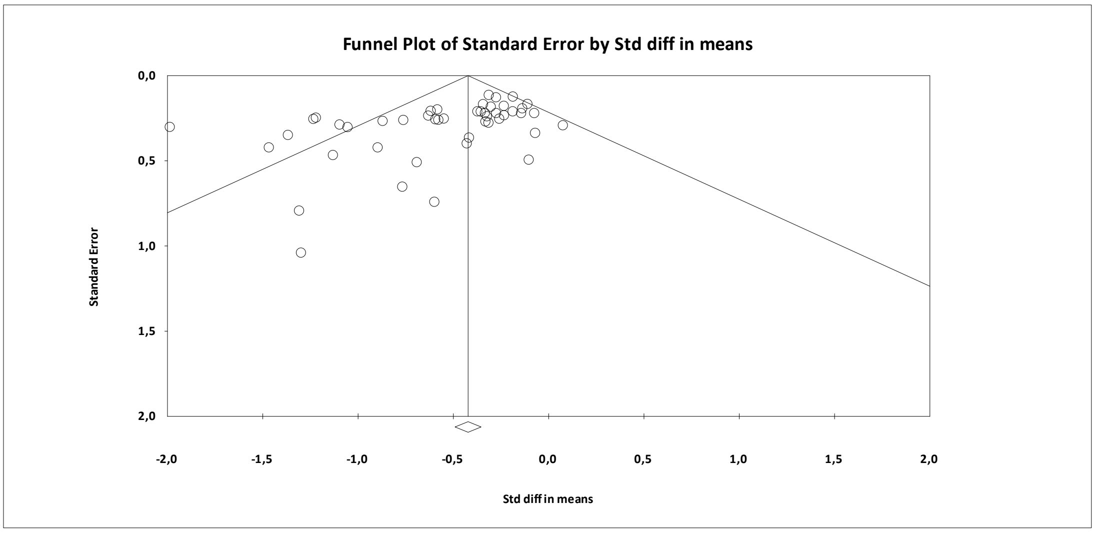

sFigure2 *Funnelplot of cognitive outcomes*

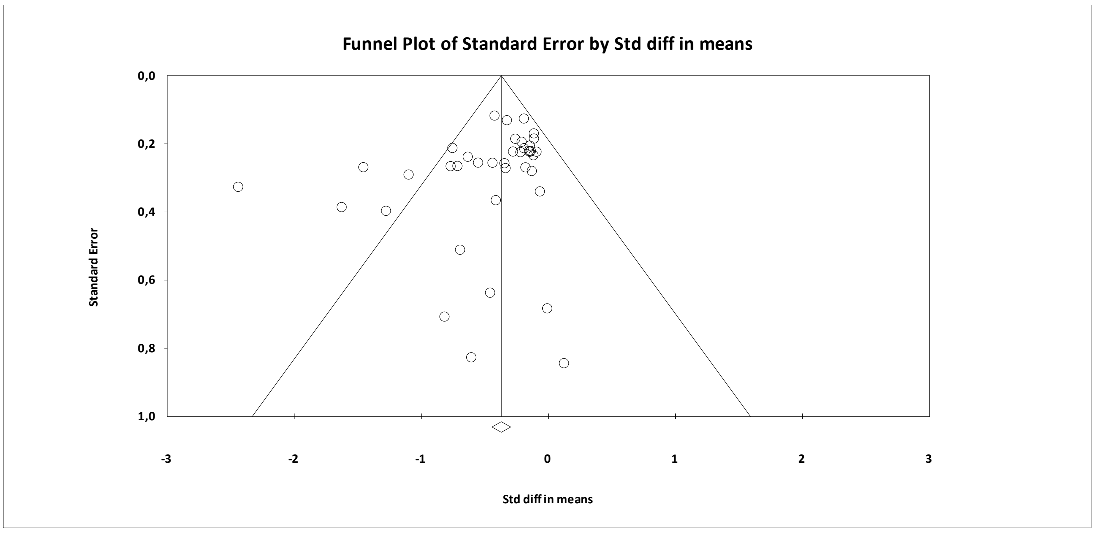

sFigure 3 *Funnelplot of motor outcomes*

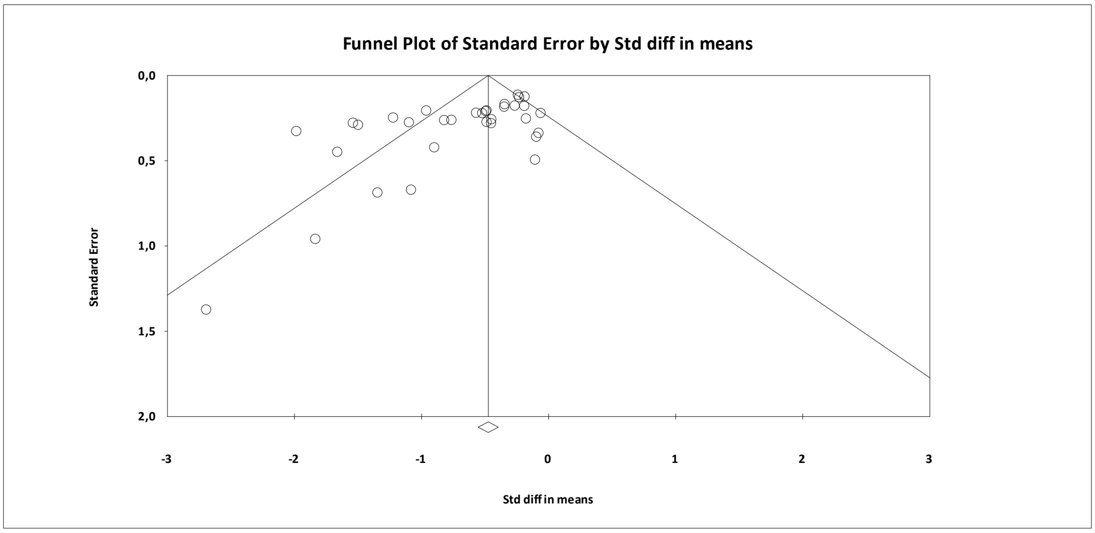

sFigure4 *Funnelplot of language outcomes*

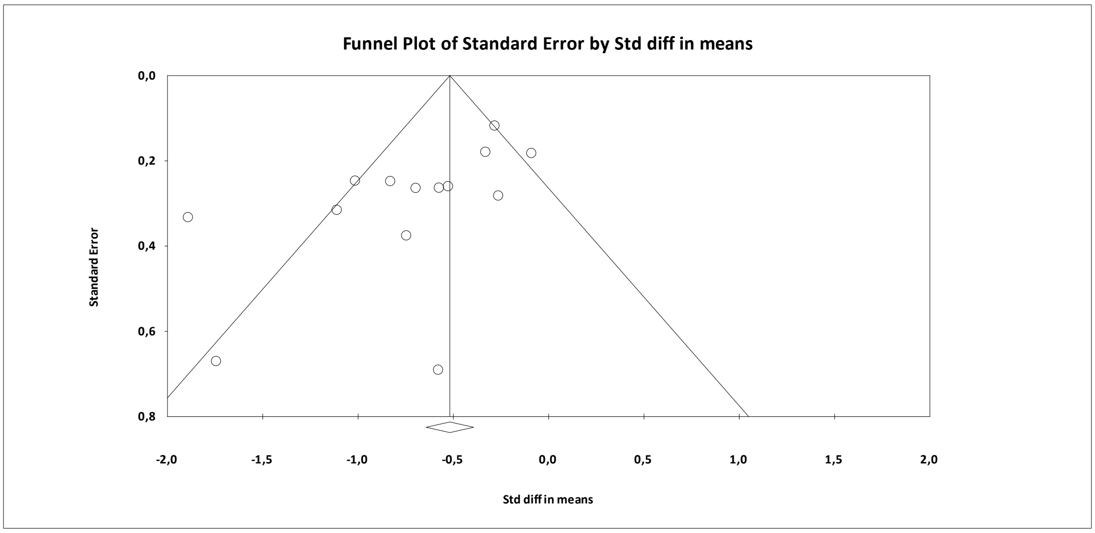

All funnel plots were symmetric on visual inspection and showed no asymmetry. However, Egger's regression showed significant risk of publication bias for all meta-analyses. Risk of potential assessment bias was found for (a) 39 of the 47 studies because these studies compared patient data with normative data standardized for age only, leaving other potentially confounding factors uncontrolled, and (b) 22 of the 47 studies because of loss to follow-up of more than 70% of the participants which may lead to a potential bias either due to loss of high-functioning patients or due to loss of severely impaired patients with co-morbidity and subsequent higher mortality.

PRISMA CHECKLIST

Section/topic	#	Checklist item	Reported on page #
**TITLE**			
Title	1	Identify the report as a systematic review, meta-analysis, or both.	1
**ABSTRACT**			
Structured summary	2	Provide a structured summary including, as applicable: background; objectives; data sources; study eligibility criteria, participants, and interventions; study appraisal and synthesis methods; results; limitations; conclusions and implications of key findings; systematic review registration number.	2-3
**INTRODUCTION**			
Rationale	3	Describe the rationale for the review in the context of what is already known.	4-5
Objectives	4	Provide an explicit statement of questions being addressed with reference to participants, interventions, comparisons, outcomes, and study design (PICOS).	5
**METHODS**			
Protocol and registration	5	Indicate if a review protocol exists, if and where it can be accessed (e.g., Web address), and, if available, provide registration information including registration number.	N/A.<br>The study protocol can be provided by authors.
Eligibility criteria	6	Specify study characteristics (e.g., PICOS, length of follow-up) and report characteristics (e.g., years considered, language, publication status) used as criteria for eligibility, giving rationale.	5-6, see Search and Selection
Information sources	7	Describe all information sources (e.g., databases with dates of coverage, contact with study authors to identify additional studies) in the search and date last searched.	5, see Search and Selection
Search	8	Present full electronic search strategy for at least one database, including any limits used, such that it could be repeated.	Supplementary Material.
Study selection	9	State the process for selecting studies (i.e., screening, eligibility, included in systematic review, and, if applicable, included in the meta-analysis).	5, see Search and Selection
Data collection process	10	Describe method of data extraction from reports (e.g., piloted forms, independently, in duplicate) and any processes for obtaining and confirming data from investigators.	6-7, see Data-extraction
Data items	11	List and define all variables for which data were sought (e.g., PICOS, funding sources) and any assumptions and simplifications made.	6-7, see Data-extraction
Risk of bias in individual studies	12	Describe methods used for assessing risk of bias of individual studies (including specification of whether this was done at the study or outcome level), and how	7-8<br>- See Quality assessment

		this information is to be used in any data synthesis.	and Statistics
Summary measures	13	State the principal summary measures (e.g., risk ratio, difference in means).	7, See Statistics: Cohen's d
Synthesis of results	14	Describe the methods of handling data and combining results of studies, if done, including measures of consistency (e.g., I^2) for each meta-analysis.	7-8, see Statistics:<br>- I^2<br>- Random-effects meta-analysis
Risk of bias across studies	15	Specify any assessment of risk of bias that may affect the cumulative evidence (e.g., publication bias, selective reporting within studies).	7-8, see Statistics:<br>- Visual inspection funneplots<br>- Eggers intercept
Additional analyses	16	Describe methods of additional analyses (e.g., sensitivity or subgroup analyses, meta-regression), if done, indicating which were pre-specified.	7-8, see Statistics:<br>Sensitivity analysis were done to compare outcomes at different domains of neurodevelopment, to compare different types of malformations and to compare evidence of different study quality.
**RESULTS**			
Study selection	17	Give numbers of studies screened, assessed for eligibility, and included in the review, with reasons for exclusions at each stage, ideally with a flow diagram.	5-6, see Search and Selection; Figure 1
Study characteristics	18	For each study, present characteristics for which data were extracted (e.g., study size, PICOS, follow-up period) and provide the citations.	Supplementary Table, sTable 1
Risk of bias within studies	19	Present data on risk of bias of each study and, if available, any outcome level assessment (see item 12).	Table 2
Results of individual studies	20	For all outcomes considered (benefits or harms), present, for each study: (a) simple summary data for each intervention group (b) effect estimates and confidence intervals, ideally with a forest plot.	Figure 2 / Table 2
Synthesis of results	21	Present results of each meta-analysis done, including confidence intervals and measures of consistency.	9-11, described in results section, Table 2
Risk of bias across studies	22	Present results of any assessment of risk of bias across studies (see Item 15).	12-13
Additional analysis	23	Give results of additional analyses, if done (e.g., sensitivity or subgroup analyses, meta-regression [see Item 16]).	11-15, Supplementary Table sTable 2
**DISCUSSION**			
Summary of evidence	24	Summarize the main findings including the strength of evidence for each main outcome; consider their relevance to key groups (e.g., healthcare providers, users, and policy makers).	16-17, summary of findings was given in the first part of the discussion

Limitations	25	Discuss limitations at study and outcome level (e.g., risk of bias), and at review-level (e.g., incomplete retrieval of identified research, reporting bias).	18, limitations were discussed under "limitations"
Conclusions	26	Provide a general interpretation of the results in the context of other evidence, and implications for future research.	18, see Conclusions
**FUNDING**			
Funding	27	Describe sources of funding for the systematic review and other support (e.g., supply of data); role of funders for the systematic review.	N/A
